# Book Reviews

**Published:** 1899-06

**Authors:** 


### Book Reviews.

The Practice of Obstetrics. By American authors. Edited by Charles Jewett, M. D., Professor of Obstetrics in Long Island College Hospital, Brooklyn, N. Y. In one 8vo volume of 736 pages, with 441 engravings in colors and black and twenty-two full-page colored plates. Philadelphia and New York. Lea Brothers & Co., Publishers. 1899.

The literature of obstetrics is further enriched and embellished by this practical work, which brings forward the teachings of the medicine and surgery of the lying-in room to the present day.

It is most important for the student of obstetrics to become well grounded in the anatomy and physiology of this special department of medicine. We speak advisedly. This is a *special* department of the science and art of medicine. Moreover, it is one of the most important departments thereof, since it nearly always deals clinically with the lives of two or more persons. Its anatomy and physiology is likewise special. For these reasons Jewett has wisely committed these branches of his subject to most capable men, two chapters thereof being written by himself.

Browning, of Brooklyn, has prepared a most comprehensive, concise and instructive chapter on the anatomy of the female pelvic organs and the mammary glands, which is profusely and beautifully illustrated. The engravings are works of art that teach the subject almost independently of the text, predisposing, of course, a general anatomical knowledge on the part of the reader.

The physiology of pregnancy forms the subject of the second part, which is dealt with in five chapters, written by Walter P. Manton, Chauncey B. Palmer and Robert L. Dickinson. The chapter on the diagnosis of pregnancy deals with a question that is sometimes very perplexing to the novice. Dr. Dickinson, who wrote this chapter, has succeeded in divesting the subject of much that is complicated in the minds of the beginner, and has thus made it very much more simple to everybody. The physiology of labor comprises the third part, of which there are three chapters, two written by the editor and one by Dr Augustus H. Buckmaster. The physiology of the puerperium constitutes the fourth part, of which there are two chapters, written respectively by Hunter Robb and Elias H. Bartley. These deal with the management of the puerperal state, the care of the puerperal woman, and the management of the new-born child—all essentials and all instructively handled.

The fifth part, devoted to the consideration of the pathology of pregnancy, consists of seven chapters, written by Walter P. Manton, James H. Etheridge, Joshua M. Van Cott, Jr., Hiram N. Vineberg and Fernand Henrotin. The various chapters are well written, as might be anticipated from the experience of the several authors. Those of this group most important to the general practitioner of obstetrics—in other words, to the family doctor—are the chapters on abortion and premature labor by Vineberg, and the diseases of pregnancy by Etheridge. These two subjects confront the general practitioner of medicine almost daily; hence he should be well versed in the most approved expedients with which to meet or avert their dangers. The other chapters are only less important because they deal with conditions and complications less frequently met.

The next, or sixth part takes up for consideration the pathology of labor and consists of six chapters. Its authors are J. Chalmers Cameron, J. Clarence Webster, Etheridge, Jewett, and J. Clifton Edgar. Here we find most competently handled the anomalies of the mechanism of the expellent forces of the passages, of the fetus, of these arising from accidents or disease, and of the hemorrhages. The chapter dealing with eclampsia, by Edgar, is a most able exposition of the subject and contains in concise form an expression of the most valuable part of our knowledge up to the present moment relating to this subtle and direful malady, upon which there are so many divergent views and disputed points. We have heretofore had occasion to speak in favorable terms of Edgar's work and position regarding eclampsia, and this chapter containing a expression of his latest views only serves to accentuate our previous good opinion of his teaching.

The pathology of the puerperium forms the subject of the seventh part, comprising four chapters written by Van Cott, Allan McLane Hamilton, J. Whitridge Williams and Henry Dwight Chapin. They deal with the anomalies of the breast and nipples (Van Cott) puerperal insanity (Hamilton) puerperal infection (Williams) and malformations, injuries and diseases of the new-born child (Chapin) There is no more important subject relating to obstetrics than puerperal infection, and it is one with which the everyday obstetrician should be thoroughly familiar. In this monograph will be found an exposition of the part microörganisms play in general and in particular, together with all external modes of infection and auto-infection, as well as a disquisition upon treatment, curative and preventative.

Finally, we are presented with the eighth part, comprising six chapters devoted to the consideration of obstetric surgery. The immediate repair of vaginal, vulvar and cervical lacerations (Robb)

constitutes the first chapter; the induction of abortion and of premature labor, including retained and adherent placenta, by the same author, form the subjects of the next chapter; the forceps (Jewett), that of the third; version and embryotomy (Davis), the fourth and fifth; and, lastly, Cesarean section, the Porro operation and symphyseotomy (Robb) form the concluding chapter of the book.

From the foregoing synopsis it will be observed that the editor has dealt with the whole subject most comprehensively, that he has chosen for the most part able and practical men to write the several chapters (most conspicuous among them himself), and with all, the publishers have brought out the book in a most agreeable style. The illustrations are largely new and original and in almost every instance they are further explanatory of the text.

Conspicuous among the contributions to this work are five chapters which bear the authorship, in whole or in part, of James H. Etheridge, of Chicago. Sadly enough they comprise his last important contribution to the literature of medicine, for he passed from life almost simultaneously with the issuance of the book from the press. These chapters, therefore, will possess an added interest to his many professional friends.

International Clinics. A Quarterly of Clinical Lectures and Specially Prepared Articles on Treatment and Drugs. By professors and lecturers in the leading medical colleges of the United States, Germany, Austria, France, Great Britain and Canada. Edited by Judson Daland, M. D. (Univ. of Penna.), Philadelphia, Instructor in Clinical Medicine and Lecturer on Physical Diagnosis in the University of Pennsylvania; Assistant Physician to the Hospital of the University of Pennsylvania, etc.; Fellow of the College of Physicians of Philadelphia. J. Mitchell Bruce, M. D., F. R. C. P., London, England, Physician to and Lecturer on the Principles and Practice of medicine at the Charing Cross Hospital. David W. Finlay, M. D., F. R. C. P., Aberdeen Scotland, Professor of Practice of Medicine in the University of Aberdeen; Physician to and Lecturer on Clinical Medicine in the Aberdeen Royal Infirmary, etc. Volume II. Eighth series. 1898. Octavo, pp. xii.—375. Philadelphia: J. B. Lippincott Co. 1899.

This volume is an interesting conclusion of the eighth series of the clinics It treats of subjects under the following several general heads: Drugs and remedial agents, treatment, medicine, neurology, surgery, gynecology and obstetrics, ophthalmology, laryngology, pharyngology, rhinology and otology and dermatology. Under these several heads are lectures by experienced teachers, who for the most part are well known to the profession of medicine. Pieëminent among the number is the name of Professor Joseph M. Mathews, of Louisville, who is this year president of the American Medical Association and who lectures upon the treatment of ulceration of the rectum and ulceration of the colon. Professor Charles Greene Cumston, of Boston, also represents an instructive lecture upon simple hydrocele and its treatment. There are a number of foreign contributions and a few of the lectures are well illustrated, especially that upon lupus vulgaris, by Dr. Norman Walker.

---

Materia Medica, Pharmacy, Pharmacology and Therapeutics. By W. Hale White, M. D., F. R. C. P., Physician to, and Lecturer on Pharmacology and Therapeutics at Guy's Hospital, London; Examiner in Materia Medica to University of London, etc., etc. Edited by Reynold W. Wilcox, M. A , M.D., LL D., Professor of Medicine and Therapeutics at the New York Post-Graduate Medical School, and Attending Physician to the Post-Graduate Hospital, etc., etc. Fourth American Edition. Thoroughly revised. Small 8vo, pp. 704. Philadelphia : P. Blakiston's Son & Co. 1898.

The fourth edition of this valuable work shows that its worth and excellence has been appreciated by the profession and in this new edition, the confidence put in the former editions may be safely continued. The manner in which the action of various drugs upon the various systems, as discussed in the book, is an admirable one, thus enabling the busy practitioner to seek the desired information in a few moments. We can only reiterate what was said in a former review of this work, that it is one of the best reference books a student can consult, and is admirably adapted to the needs of the general practitioner of medicine.

In make-up it is very similar to former editions and in the arrangement of the various drugs is more convincing than heretofore.

The page containing the press notices of the previous American editions, just preceding the title page, might with propriety have been placed after the index, or preferably omitted altogether.

---

Transactions of the American Gynecological Society, Vol. XXIII., for the year 1898. J. Riddle Goffe, M. D., Secretary. Small 8vo, pp. 491. Philadelphia: Wm. J. Dornan, Printer. 1898.

The annual volume of the transactions of this society is always a welcome addition to the literature of gynecological medicine. One of the most interesting features of this book is a discussion, entitled, Has electricity ceased to be a useful therapeutic agent in gynecology? Two papers were presented favoring the negative of the question as propounded, and one taking the affirmative side. The latter was presented by Dr. Egbert H. Grandin, of New York, and is an admirably and manly setting forth of a change of view which has possessed the author of late years. During the course of his remarks we observe the following : "Five years ago my electrical outfit was an elaborate one; to-day I possess no outfit, having given it away to a colleague whose credulity was born as mine died." This is practically the attitude of the most progressive men with regard to this mysterious agent.

At the end of the book are printed obituary notices, first, of William Thompson Lusk by Thaddeus A. Reamy; second, of Cornelius Kollock by Richard B. Maury; third, of John Braxton Hicks by Paul F. Mundé; fourth, of Professor S. Tarnier by A. Charpentier; and fifth, of Henry Parke Curtis Wilson by B. Bernard Browne. These are all accompanied by excellent portraits of these distinguished men.

ESSENTIALS OF MATERIA MEDICA, THERAPEUTICS AND PRESCRIPTION WRITING. Arranged in Form of Questions and Answers. Especially for Students of Medicine. By HENRY MORRIS, M. D., Physician to St. Joseph's Hospital, etc. Fifth edition, revised and enlarged. Philadelphia: W. B. Saunders, 925 Walnut Street.

This little book needs no better recommendation than that a fifth edition is in demand. It has undergone a thorough revision, much that was obsolete being omitted and many new drugs, especially of the hypnotic and antipyretic class, have been added. The dosage of each preparation is given both in apothecaries' and metric measure. The classification of remedies is based on a therapeutic method, perhaps the most rational plan that can be adopted, and one which will serve to recall to the student when he shall have left the class-room for the actual practice of medicine, the various remedies which the case he has in hand may demand.

---

THIRTEENTH ANNUAL REPORT of the State Board of Health of Pennsylvania for the year 1897. In two volumes. Benjamin Lee, M. D., Secretary. Wm. Stanley Ray, State Printer. 1898.

The accomplished secretary of the Pennsylvania state board of health is entitled to great credit for the manner in which these reports are published. Pennsylvania after the fashion of Michigan and perhaps some other states, has sanitary conferences that are appointed to be held in different sections of the state, the proceedings of which are printed in these reports. These conferences are not simply conventions of public health officials, but are open to attendance on the part of the general public who are invited to participate in the proceedings.

This report contains an excellent drawing of the Conemaugh Valley, showing the vicinity of Johnstown, the scene of the mighty flood of ten years ago. Besides this, there are other excellent illustrations in half-tones and colors that add to the interest of the volumes.

Three Thousand Questions on Medical Subjects. Arranged for self-examination with the proper reference to standard works in which the correct replies will be found. Second edition, enlarged. Philadelphia: P. Blakiston's Son & Co. 1899.

This book is one of those ingenious contrivances for the torture of the student who is preparing himself for examination. There is a better way to prepare for examinations than that afforded by the use of quiz compends and questions for self-examination, but for those who have never learned it these things serve a useful purpose.

---

A Syllabus of Materia Medica. Compiled by Warren Coleman, M. D,, Instructor in Clinical Medicine and Materia Medica in Cornell University, Medical Department. One volume. 16mo, 175 pages. Polished buckram. New York: William Wood & Co. 1899.

This little volume will prove an excellent help to the student in study of materia medica. The actions and uses of drugs, dosage, dosage in children, incompatibilities, and prescription writing are all presented in such manner as to appeal to the memory. But one criticism is to be made on the volume, and that is that dosage is not given in the metric as well as in the common system.

---

A Text-Book on Practical Obstetrics. By Egbert H. Grandin, M. D., Gynecologist to the Columbus Hospital; Consulting Gynecologist to the French Hospital; Late Consulting Obstetrician and Obstetric Surgeon of the New York Maternity Hospital, etc. With the collaboration of George W. Jarman, M. D., Gynecologist to the Cancer Hospital; Instructor in Gynecology in the Medical Department of the Columbia University. Second edition; revised and enlarged. Illustrated; pp. xiv.—461. Philadelphia: The F. A. Davis Co. 1899.

This book in its second edition presents so much that is new that a somewhat detailed review is called for. It differs from older and larger treatises on obstetrics in that it assumes the reader to possess a sufficient knowledge of anatomy, physiology, pathology and embryology, and no attempt is made, therefore, to include these subjects in the work. Facts relating to them are only given when a proper understanding of the text demands them.

The volume is divided into four parts. Part I. deals with pregnancy, taking up the question of diagnosis, differential diagnosis, duration and hygiene of pregnancy, the pathology of pregnancy, and the diagnosis of presentation and position. In this section, as well as throughout the book, one notices the excellence of the illustrations. Those which are borrowed from older works on obstetrics are only such as can not be improved upon as elucidators of the text. The sixty-four full-page photographic plates, original with the authors, increase many fold the value of the book. Part II. discusses the subject of labor. The difficult topic of the mechanism of labor is presented with unusual clearness. Indeed, we rarely, if ever, have seen it better handled. The clinical course of labor, the management of normal and abnormal labor, and the care of the

ANNUAL AND ANALYTICAL CYCLOPEDIA OF PRACTICAL MEDICINE. By CHARLES E. DE M. SAJOUS, M. D., and 100 associate editors. Assisted by corresponding editors, collaborators and correspondents. Illustrated with chromo-lithographs, engravings and maps. Volume III. Dislocations to Infantile Myxedema. Philadelphia, New York and Chicago: F. A. Davis Company. 1899.

As these volumes continue to issue from the press their importance to medical literature seems to grow upon the observer. This third volume carries the work forward to about the middle of the alphabet, hence the series is half completed. The staff of associate editors is a strong one and each has performed his work with exceptional ability. The editor-in-chief very properly expresses his obligations to these gentlemen for their efficient work. It is not always easy to obtain efficient collaboration in a work so extensive as this and we think the editor-in-chief deserves the thanks of the profession upon his success in that regard.

There are a number of articles of more than usual interest in this volume, among which may be mentioned dysentery, endometritis, dislocations and fractures, gout, hip-joint diseases, eczema, hysteria and hypnotism. This volume is also quite elaborately illustrated, which, of course, adds materially to its value.

---

A LABORATORY GUIDE TO URINALYSIS AND TOXICOLOGY. By R. A. WITTHAUS, M. D., Professor of Chemistry, Physics and Toxicology in the Medical Department of Cornell University; Professor of Chemistry and Toxicology in the Medical Department, University of Vermont, etc., etc. Fourth edition. New York: William Wood & Co. 1898.

This practical handbook having already gone through several editions has become so established as a favorite for student and physician, that very little can be said in regard to it beyond that which has already been written.

The author has been a practical teacher so many years that he well knows the needs of the laboratory worker and it requires but little examination of this guide to satisfy one of its practical nature.

The rules were numbered consecutively from 1 to 440. Each alternate page is left blank for additions and annotations, and a concise index includes the book.

Urinary Analysis and Diagnosis by Microscopical and Chemical Examinations By Louis Heitzmann, M. D., New York. One volume, 270 pages, 8vo, 108 original illustrations. New York; William Wood & Co. 1899.

Although there appears to be no long felt want for another text-book on this subject, nevertheless the author has been guided by the fact that microscopical examination and especially microscopical diagnosis have not received as much attention in the text-book as their importance calls for. Many years of experience and teaching have taught him that correct diagnosis by means of microscopical examination of urine can frequently be made in cases where chemical analysis is of little value.

This book is divided into three parts. First, chemical examination; second, microscopical examination; and third, microscopical diagnosis.

It is evident that a mere description of the features found in different cases cannot be sufficiently clear, but illustrations made directly from nature are absolutely essential. In this volume all the illustrations—108 in number—without exception, have been drawn by the author directly from specimens in his possession, and are drawn on such a scale as to be perfectly clear and comprehensible. To those who are not content with merely a chemical examination of the urine, but desire a microscopical examination as well, this work is to be recommended.

A Manual of Physiology, with Practical Exercises. By G. N. Stewart, M. A., M. D., Edin.; Professor of Physiology in the Western Reserve University, Cleveland. With numerous illustrations, including five colored plates. Third edition. Small 8vo, pp. 848. Philadelphia: W. B. Saunders. 1898.

The two previous editions of this book having been exhausted and there being a demand for a third, the author has taken advantage of the fact to revise and partly rewrite his book. It differs from most works on physiology in the introduction of practical exercises, which the author thinks he has found useful in teaching physiology. He has taken pains to note that all experiments on animals which would cause pain have been done under anesthesia. The volume contains a number of useful illustrations, the plates in color being especially well executed. This manual is a useful book for the beginner.

TRANSACTIONS OF THE TEXAS STATE MEDICAL ASSOCIATION. Thirtieth Annual Session, held at Houston, Texas, April 26, 27, 28 and 29, 1898. Edited by H. A. WEST, M. D., Secretary. Austin, Texas. 1898.

Transactions of this association year by year show improvement through the guiding hands of a masterful secretary, supported by other skilful coadjutors.

The association groups its work into sections, the first relating to general medicine; the second, to obstetrics and diseases of children ; the third, to surgery; the fourth, to gynecology ; and the fifth, to ophthalmology and otology. The average number of papers in each section is six, and the titles are so distributed as to cover each separate field in a practical manner. The volume is well printed and handsomely bound.

